# The effect of lycopene supplementation on radiation-induced micronuclei in mice reticulocytes in vivo

**DOI:** 10.1007/s00411-019-00795-0

**Published:** 2019-05-23

**Authors:** Małgorzata M. Dobrzyńska, Aneta Gajowik, Joanna Radzikowska

**Affiliations:** Department of Radiation Hygiene and Radiobiology, National Institute of Public Health – National Institute of Hygiene, 24 Chocimska Street, 00-791 Warsaw, Poland

**Keywords:** Irradiation, Radiomitigation, Radioprotection, Lycopene, DNA damage, Micronuclei

## Abstract

Lycopene (LYC) is a natural pigment present in tomatoes and other red fruits and vegetables including red carrots, red peppers, watermelons, pink grapefruits, apricots, pink guavas, and papaya. There is some evidence that LYC may provide protection against mutations induced by ionizing radiation. The study aimed to investigate whether the genetic material of reticulocytes (RET) could be protected from radiation-induced damage by LYC. Mice were treated with LYC [0.15 mg/kg bodyweight (bw), 0.30 mg/kg bw], acute and fractionated irradiation (0.5 Gy, 1 Gy applied daily), or with both agents (0.5 Gy + 0.15 mg/kg bw LYC, 0.5 Gy + 0.30 mg/kg bw LYC, 1 Gy + 0.15 mg/kg bw LYC, 1 Gy + 0.30 mg/kg LYC). LYC supplementation was started at 24 h or 1 week after the first irradiation. Irradiation significantly enhanced the frequency of micronuclei (MN) in RET. LYC treatment at a dose of 0.15 mg/kg bw 24 h after starting fractionated radiation at 1 Gy significantly decreased (41–68%, *p* < 0.0125) the level of MN in peripheral blood and bone marrow RET. LYC supplementation at 0.30 mg/kg bw did not significantly alter the frequency of MN in peripheral blood, but significantly increased the frequency of bone marrow RET MN. LYC treatment on day 8 following the first radiation exposure showed results similar (92–117%, *p* > 0.24) to those obtained with irradiation alone. Lycopene may act as a radiomitigator but must be administered at low doses and as soon as possible after irradiation. Contrary, combined exposure with high doses of irradiation and LYC may enhance the mutagenic effect of irradiation.

## Introduction

Ionizing radiation (IR) is continuously present in the natural environment and at various human workplaces. Natural background radiation comes from cosmic plus solar radiation and terrestrial radionuclides that occur in the Earth’s crust, building materials, air, water, food and the human body. The dose of naturally occurring radiation, i.e., the background effective dose is estimated to be ~ 2.4 mSv per year, and about 3 mSv including artificial sources (UNSCEAR 2008). IR may be emitted during the natural decay process of certain unstable nuclei, or following the excitation of atoms and their nuclei in nuclear reactions, cyclotrons, X-ray machines, and others including medical devices. Moreover, there are occupations where people are exposed to radiation such as airline crews; industrial radiography, medical radiology, and nuclear medicine staff; uranium mining workers; nuclear power plant and nuclear fuel reprocessing plant workers; and research laboratory employees (UNSCEAR 2008; Pattison et al. 1996; Pattison 1999; Hendry et al. 2009; Eisenbud 1987).

IR represents electromagnetic waves and particles that carry sufficient energy to ionize or remove electrons from an atom. Ionization provoked by radiation induces several chemical reactions leading to serious changes in atoms and molecules, which may thus lead to cell damage. There are two primary mechanisms of IR interactions with biological matter: direct effects owing to deposition of energy with a macromolecule, and indirect effects from the interaction of energy with water to produce reactive oxygen species (ROS) (Barcellos-Hoff et al. 2005). For both types of electromagnetic waves that can ionize atoms, i.e., X-rays and γ-rays, 60% of the damage is caused by indirect effects. The impact of IR is a function of the physical attributes of radiation type, dose, and whether the exposure is acute, fractionated, or chronic. Biological responses to radiation depend on the age, tissue type, genetic background, and physiological status of the exposed individual (Barcellos-Hoff et al. 2005 Desouky et al. 2015; Ward 1988).

Molecules with direct free-radical-scavenging properties are particularly promising in protection against radiation. The discovery of non-toxic modifiers/radioprotectors (agents which when present prior to or shortly after radiation exposure, alter the tissue responses to radiation) and/or radiomitigators (agents that may be used to minimize toxicity even when applied after radiation exposure) is very important for radiation protection of humans (Cirin et al. 2010). Dietary antioxidants may protect cells against DNA damage induced by endogenous and exogenous sources, including IR. Carotenoids are organic lipid-soluble pigments found in the chloroplasts and chromoplasts of plants (Krzyzanowska et al. 2010). Lycopene (LYC) is an acyclic isomer of β-carotene and a natural pigment, synthesized by plants and microorganisms. It is the main carotene present in tomatoes and other red fruits and vegetables including red carrots, red peppers, watermelons, pink grapefruits, apricots, pink guavas, and papaya (Islamian and Mehrall 2015). LYC concentrations in tomatoes range from 7.8 to 18.1 mg/100 g fresh weight (fw) (Martí et al. 2016), whereas in tomato paste, they range from 51 to 59.7 mg/100 g (Tonucci et al. 1995). LYC is one of the most potent antioxidants, with singlet oxygen-quenching ability twice as high as that of β-carotene and 10 times higher than that of α-tocopherol (Di Mascio et al. 1989).

The antioxidative, radioprotective and anticancer properties of LYC were reviewed by Gajowik and Dobrzyńska (2014). Evidence for the ability of lycopene to modulate ROS levels showed that it may chemically interact with ROS and undergo oxidation, thus preventing ROS-induced cell damage (Palozza et al. 2010). Previous studies have suggested that inclusion of LYC in diet can reduce the risk of cancer (Giovannucci 1999; Rao and Agarwal 2000) and cardiovascular diseases (Rao and Agarwal 2000; Rao 2002).

There is some evidence that LYC may provide protection against mutations induced by ionizing radiation. Numerous studies have shown the beneficial effect of LYC when administered before or during irradiation (Saada and Azab 2001; Srinivasan et al. 2007; 2009; Cavusoglu and Yalcin 2009; Saada et al. 2010). In contrast, there are few papers describing the effect of LYC administered after irradiation (Forssberg et al. 1959; Maydan et al. 2011).

The present study aimed to investigate whether protection of the genetic material of reticulocytes (RET) from damage expressed as radiation-induced micronuclei, could be achieved by co-administration of LYC at different times after irradiation.

## Materials and methods

### Animals and exposure to radiation and LYC

**Fig. 1:**
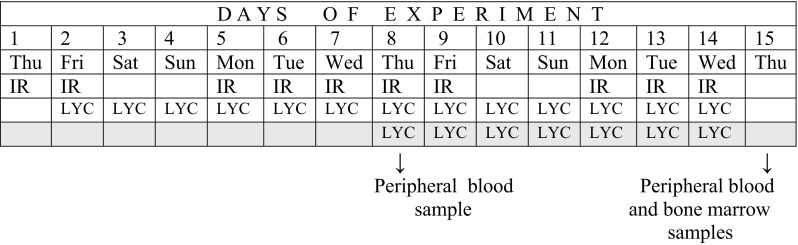
Experimental design for timing of irradiation, LYC supplementation and sampling time for LYC supplementation beginning 24 h after irradiation (white background) or LYC supplementation beginning eighth day after irradiation (gray background)

Seven-week-old male Swiss outbred laboratory mice obtained from the “Kołacz” Animal Breeding Laboratory (Warsaw, Poland) were housed in standard rodent cages in a room with controlled temperature, humidity, and light cycles (12 h dark, 12 h light). Tap water and rodent diet were available ad libitum. The mice were randomly assigned to either control or exposed groups, 1 week after acclimatization. LYC (ROTH cat. no: 1180.1, purity > 90%) was dissolved in 1 ml of DMSO and diluted in drinking water to obtain the desired dose. The concentration of DMSO in drinking water with LYC was less than 0.25%. 8-week-old male mice were exposed to LYC (0.15 mg/kg body weight (bw) or 0.30 mg/kg bw, daily), irradiation with X-rays (0.5 Gy or 1 Gy, daily), or a combination of both agents (0.5 Gy + 0.15 mg/kg bw LYC, 0.5 Gy + 0.30 mg/kg bw LYC, 1 Gy + 0.15 mg/kg bw LYC, 1 Gy + 0.30 mg/kg bw LYC daily). Animals were irradiated 5 times per week (working days), whereas LYC was continuously supplied in drinking water starting from 24 h or 1 week following the initiation of daily irradiation. Body weight of the animals was checked weekly, and the volumes of control water and LYC-water solution were checked daily. The solution of LYC in water was prepared twice a week. A therapeutic Roentgen unit Medicor type THX-250 (Hungary) was used for irradiation. It was operated with the following parameters: 155 kV, 18 mA, added filtration 0.25 mm Cu and HVL 2 mm Al. Mice were subjected to whole-body irradiation at the dose rate of 0.2 Gy/min. In the case of combined exposure, LYC supplementation was started at 24 h or 1 week (8th day) *following initial radiation exposure* (Fig. 1). Control animals were sham irradiated and unexposed to LYC, and mice exposed only to LYC. Mice from above groups and from the control group received tap water. Animals were killed 24 h after the last irradiation. Six to eight mice were used for each of the dose and time period. The total doses to mice where LYC supplementation started 24 h following radiation were 5 Gy or 10 Gy for irradiation, 1.95 mg/kg bw or 3.9 mg/kg bw for LYC, and 5 Gy + 1.95 mg/kg bw LYC, 5 Gy + 3.9 mg/kg bw LYC, 10 Gy + 1.95 mg/kg bw LYC, and 10 Gy + 3.9 mg/kg bw LYC for combined exposure. The total doses to mice where LYC supplementation started at 1 week following irradiation were 5 Gy or 10 Gy for irradiation, 1.05 mg/kg bw or 2.1 mg/kg bw for LYC, and 5 Gy + 1.05 mg/kg bw LYC, 5 Gy + 2.1 mg/kg bw LYC, 10 Gy + 1.05 mg/kg bw LYC, and 10 Gy + 2.1 mg/kg bw LYC for combined exposure.

The study was approved by the IV Local Ethical Commission for Animal Experiments.

### Micronucleus test

For the micronucleus (MN) assay, blood samples were drawn from the caudal vein at 1 (day 8) and 2 weeks (day 15) after the first irradiation. Bone marrow samples were harvested from animals killed at 2 weeks after the treatment. Femoral bone marrow cells were aspirated with 3 ml fetal bovine serum. Cells were centrifuged at 800×*g* for 10 min, and after removing the majority of the supernatant, the pellet was resuspended. For examination of the MN in reticulocytes (RET), the method described by Hayashi et al. (1990) was adapted. Cells in suspension (25 µl) were dropped on microscope slides coated with acridine orange solution and immediately covered with a cover slip. Similarly, 10 µl of peripheral blood was dropped on a microscope slide coated with acridine orange solution followed by covering with a cover slip. Using a fluorescence microscope (Nikon, Japan), one thousand reticulocytes per animal were scored from the bone marrow and peripheral blood cells to calculate micronucleus frequencies. The MN-PCE frequencies were calculated as (total number of MN scored/1000 PCEs) × 100 and reported as MN-PCE% (mean pooled value for all mice in each group ± standard deviation.

### Statistical analysis

The data were analyzed by one-way analysis of variance (ANOVA), with a post hoc Fisher test. The significance level was set at *p* < 0.05.

## Results

The results of LYC supplementation beginning 24 h after the first irradiation on the MN frequency in mouse RET are shown in Table 1. At the beginning of the experiment the mean body weight of mice from the group exposed to 0.5 Gy of irradiation daily was significantly higher compared to control. The mean body weights of animals of groups 0.5 Gy + 0.15 mg/kg bw LYC, 1 Gy + 0.15 mg/kg bw LYC and 1 Gy + 0.30 mg/kg bw LYC were significantly lower compared to animals exposed to appropriate dose of irradiation. After 1 week of exposure, the body weights of animals of 1 Gy + 0.15 mg/kg bw LYC and 1 Gy + 0.30 mg/kg bw LYC were significantly lower compared to negative control and 1 Gy group alone. After 2 weeks of exposure, the mean body weights of animals of groups 0.5 Gy, 1 Gy, 0.5 Gy + 0.15 mg/kg bw LYC, 0.5 Gy + 0.30 mg/kg bw LYC, 1 Gy + 0.15 mg/kg bw LYC and 1 Gy + 0.30 mg/kg bw LYC were significantly decreased compared to control. In the case of group and 1 Gy + 0.30 mg/kg bw LYC the mean body weight was significantly higher compared to the group of 1 Gy alone.

**Table 1:** Frequency of MN (mean ± SD) in reticulocytes of mice supplemented with lycopene (LYC) since 24 h after the start of irradiation

Daily dose	Number of animals	Body weight at the beginning of experiment	Body weight in the middle of experiment (1 week)	Body weight at the end of experiment (2 week)	Peripheral blood—1 week	Peripheral blood—2 weeks	Bone marrow—2 weeks
Control	7	33.65 ± 2.16	34.28 ± 1.01	35.59 ± 2.24	4.00 ± 2.00	5.42 ± 2.88	2.63 ± 1.41
0.5 Gy	7	37.30 ± 0.75*	35.00 ± 3.61	31.63 ± 3.52*	32.86 ± 12.16***	41.14 ± 16.67***	25.17 ± 10.61*
1 Gy	8	36.53 ± 1.22	33.5 ± 4.51	22.20 ± 3.40***	58.50 ± 19.65***	49.13. ± 20.55***	72.13 ± 27.72***
0.15 mg/kg LYC	6	34.48 ± 2.56	32.68 ± 1.60	33.06 ± 2.27	3.17 ± 1.33	5.13 ± 2.30	2.38 ± 1.30
0.30 mg/kg LYC	6	32.47 ± 2.59	33.94 ± 0.86	34.11 ± 2.26	3.34 ± 2.42	5.17 ± 2.93	1.38 ± 1.19
0.5 Gy + 0.15 mg/kg LYC	8	33.20 ± 2.10^b1^	32.16 ± 1.01	31.56 ± 2.19**	32.38 ± 17.82***	44.38 ± 16.80***	44.13 ± 12.60***
0.5 Gy + 0.30 mg/kg LYC	7	32.20 ± 2.22	31.50 ± 2.13	30.95 ± 1.71***	26.00 ± 20.77*	45.57 ± 19.31***	42.86 ± 16.01***
1 Gy + 0.15 mg/kg LYC	8	31.01 ± 2.64^a2^	29.82 ± 1.38**^,a2^	23.01 ± 2.39***	24.00 ± 8.11*^,a2^	36.67 ± 18.37***	49.25 ± 30.78***^,a1^
1 Gy + 0.30 mg/kg LYC	8	31.86 ± 4.48^a1^	30.00 ± 4.24*^,a1^	26.62 ± 4.49***^,a2^	60.88 ± 20.99***	64.71 ± 24.47***	95.38 ± 23.61***^,a3^

*p < 0.05 compared to control; **p < 0.01 compared to control; ***p < 0.001 compared to control

^a1^*p* < 0.05 compared to 1 Gy alone; ^a2^*p* < 0.01 compared to 1 Gy alone; ^a3^*p* < 0.001 compared to 1 Gy alone; ^b1^*p* < 0.05 compared to 0.5 Gy alone; ^b2^*p* < 0.01 compared to 0.5 Gy alone; ^b3^*p* < 0.001 compared to 0.5 Gy alone; by post hoc Fisher’s test

Two weeks of LYC treatment with doses of 0.15 mg/kg bw or 0.30 mg/kg bw daily did not induce MN frequencies higher than those in control mice. Daily irradiation of animals with 0.5 Gy as well as 1 Gy induced a 7.5–27.5-fold increase in the frequency of MN in RET from both peripheral blood and bone marrow compared to the controls. Compared to radiation alone, LYC supplementation (0.15 mg/kg bw, 0.30 mg/kg bw) simultaneously with irradiation at a daily dose of 0.5 Gy did not change the frequency of MN in peripheral blood RET measured at 1 week post-irradiation in peripheral blood or in bone marrow RET measured at 2 weeks post-irradiation. LYC administration at 0.15 mg/kg bw beginning 24 h after irradiation with 1 Gy significantly decreased the level of radiation-induced MN in peripheral blood RET at the first week post-irradiation and in the bone marrow by the end of exposure, but not in peripheral blood after 2 weeks of treatment (Table 1). LYC supplementation at the dose of 0.30 mg/kg bw did not significantly change the frequency of radiation-induced MN in peripheral blood, but significantly increased the MN frequency in bone marrow RET after the end of 1 Gy exposure.

The effects of LYC supplementation on MN induction in mouse RET beginning on day 8 after the first irradiation are shown in Table 2. At the beginning of the experiment, the mean body weights of mice from groups 1 Gy and 1 Gy + 0.15 mg/kg bw LYC were significantly lower, whereas from group 0.15 mg/kg bw LYC was significantly higher as compared to controls. After 1 week of exposure, the mean body weight of animals from the 0.1 Gy group was significantly lower as compared to the control group. After 2 weeks of exposure the body weights of mice from groups 1 Gy, 1 Gy + 0.15 mg/kg bw LYC and 1 Gy + 0.30 mg/kg bw LYC were significantly reduced compared to control. Additionally, the mean body weight of animals from the group 1 Gy + 0.30 mg/kg bw LYC was significantly different compared to animals exposed to 1 Gy alone.

**Table 2:** Frequency of MN per 1000 cells (mean ± SD) in reticulocytes of mice supplemented with lycopene (LYC) since eighth day after the start of irradiation

Daily dose	Number of animals	Body weight at the beginning of experiment	Body weight in the middle of experiment (1 week)	Body weight at the end of experiment (2 week)	Peripheral blood—1 week	Peripheral blood—2 weeks	Bone marrow—2 weeks
Control	7	33.22 ± 4.57	33.34 ± 4.49	33.90 ± 3.53	5.43 ± 2.15	4.88 ± 2.10	3.00 ± 2.00
0.5 Gy	8	30.67 ± 1.53	30.92 ± 4.34	30.17 ± 1.14	34.56 ± 7.55**	43.25 ± 8.48***	30.88 ± 13.77*
1 Gy	8	28.67 ± 1.01*	28.06 ± 3.39*	22.53 ± 1.63***	67.11 ± 30.76***	52.00 ± 20.08***	62.88 ± 30.73***
0.15 mg/kg LYC	7	37.82 ± 1.63**	37.68 ± 1.52	36.28 ± 3.79	4.71 ± 3.99	4.57 ± 2.30	2.14 ± 1.57
0.30 mg/kg LYC	7	35.88 ± 4.00	35.78 ± 3.93	34.94 ± 3.72	5.43 ± 2.76	5.00 ± 2.52	2.43 ± 1.62
0.5 Gy + 0.15 mg/kg LYC	6	30.07 ± 1.33	30.17 ± 1.36	31.84 ± 3.02	31.00 ± 20.10*	28.67 ± 18.73**	50.00 ± 19.33***
0.5 Gy + 0.30 mg/kg LYC	6	30.44 ± 1.84	31.12 ± 2.03	31.21 ± 2.43	39.67 ± 25.71***	46.00 ± 15.81***	49.71 ± 31.63***
1 Gy + 0.15 mg/kg LYC	7	28.73 ± 1.06*	30.72 ± 4.25	23.29 ± 2.05***	55.57 ± 19.41***	50.33 ± 26.64***	64.13 ± 38.23***
1 Gy + 0.30 mg/kg LYC	8	30.20 ± 1.56	30.02 ± 4.77	26.55 ± 3.72***^,a1^	61.75 ± 17.75***	61.83 ± 22.00***	60.88 ± 24.98***

*p < 0.05 compared to control; **p < 0.01 compared to control; ***p < 0.001 compared to control

^a1^*p* < 0.05 compared to 1 Gy alone; by post hoc Fisher’s test

As mentioned above, LYC administration for 1 week did not induce MN frequencies higher than those of control mice. The frequencies of MN in peripheral blood and bone marrow RET were significantly increased in both irradiated groups. In mice receiving LYC supplementation at doses of 0.15 mg/kg bw and 0.30 mg/kg bw beginning from the second week of irradiation, the MN frequencies at the end of the second week in the combined groups (i.e., LYC + irradiation) were similar to those observed after irradiation alone.

## Discussion

In the present study mice were exposed to a combination of fractionated X-radiation and LYC. Total doses of 5 Gy and 10 Gy applied within 2 weeks, i.e., 0.5 Gy and 1 Gy per day, were chosen for this study. According to the 2007 Recommendations of the International Commission on Radiological Protection, the effective dose for occupational exposure is recommended to be 20 mSv/year, averaged over 5-year periods (100 mSv in 5 years), with further provision that the effective dose should not exceed 50 mSv in a single year. Under unusual circumstances, the dose for such exposure is enhanced to 100 mSv (Annals of the ICRP 2007). Based on the precautionary principle, a factor of 10 is typically applied when extrapolating results on radiation-induced health effects from animal models to humans (Calabrese and Cook 2005). Also, a factor of 10 is applied to account for intra-individual variability in a population (Calabrese and Cook 2005). In this way, 50 mSv × 10 × 10 = 5 Sv and 100 mSv × 10 × 10 = 10 Sv. Given the fact that for X-rays and gamma rays, the equivalent dose of 1 Sv = 1 Gy and that mice were whole-body irradiated, it can be assumed that the total doses of 5 Gy and 10 Gy chosen in this animal study correspond to effective doses to humans of 50 mSv and 100 mSv, respectively, given in a short period of time.

The majority of commercially available diet supplements contain LYC at concentrations of 10 or 20 mg per tablet. For adult people weighing approximately 70–75 kg, 1 tablet corresponds to a dose of approximately 0.15 mg/kg bw or 0.30 mg/kg bw, respectively. Therefore, these doses were used in our study. We observed that one mouse drinks approximately 7 ml of water daily. We thus calculated the LYC concentration in the water for each mouse based on the above volume and its body weight. Whole-body irradiation and LYC exposure in drinking water (consumption) were chosen because these routes are the most frequent routes for human treatment.

Exposure to ionizing radiation, depending on the total dose, dose rate, and species may cause damage to living tissues, leading to induction of mutations, cancer, or cell death. In the case of lethal mutations, potentially dangerous cells are eliminated from the organism (Rodriguez-Rocha et al. 2011). Misrepaired damage may result in chromosomal damage or mutations, or cause acute adverse effects within hours to weeks or delayed effects within months to years after exposure. The resulting modification may be transmitted to further generations of cells and may eventually lead to cancer (Elmore et al. 2006; Sowa et al. 2006; Suit et al. 2007; Rodriguez-Rocha et al. 2011). IR may damage cells of the bone marrow by exhaustion of stem cells, progenitor cells, and precursor cells or by inducing genotoxic effects (Sun et al. 2011). IR induces oxidative stress through ROS generation resulting in the imbalance of pro-oxidants and antioxidants in cells (Atessahin et al. 2006).

Formation of micronuclei is associated with cytogenetic damage induced by clastogenic agents that cause disruption or breakage of chromosomes, leading to sections of the chromosome being deleted, added, or rearranged, or by aneugenic agents that interfere with the mitotic spindle apparatus (Yamamoto and Kikuchi 1980; Rosefort et al. 2004). In the present study, fractionated doses of 0.5 and 1 Gy administered to mice for 1 or 2 weeks induced MN both in the bone marrow and in peripheral blood RET. Induction of micronuclei in reticulocytes or polychromatic erythrocytes in mouse bone marrow or peripheral blood in vivo following irradiation were also observed previously at different doses and times of irradiation (Gandhi 2013; Bannister et al. 2016; Dobrzyńska et al. 2016). The frequency of micronuclei was similar for 0.5 Gy and lower for 1 Gy as compared to our earlier study, where the same doses of irradiation were used (Dobrzyńska et al. 2016).

LYC was administered in an all-*trans* form, which is its natural, stable form. LYC is unstable when exposed to light, heat, and oxygen. To prevent isomerization and oxidation, synthetic lycopene is kept under inert gas in lightproof containers and stored in a cool place (FAO 2006). The results of Nishino et al. (2013) showed that the stability of lycopene in aqueous solution was influenced by the type and amount of emulsifier used. Therefore, LYC was dissolved in DMSO, which extended its stability. The results of the present study confirmed previous results demonstrating that lycopene alone is not genotoxic, and does not induce MN in RET of the peripheral blood and bone marrow in mice in vivo (Velmurugan et al. 2004; Banji et al. 2013).

Pre-treatment of irradiated mice with tomato extract resulted in a significant reduction of chromosomal aberration frequency in the bone marrow in vivo (Dhirhe et al. 2011). Based on a study on consumption of tomatoes and tomato puree, Riso et al. (1999) suggested that consumption of tomato products may reduce the susceptibility of lymphocyte DNA to oxidative damage. In the case of LYC pre-treatment, irradiated lymphocytes are protected against DNA damage via decreased lipid peroxidation leading to improved antioxidant status (Wertz et al. 2004). It was postulated that during singlet oxygen quenching, energy is transferred from the singlet oxygen to the LYC molecule, converting it to an energy-rich triplet state (Wertz et al. 2004). Pre-treatment with 1, 5, and 10 µg/ml LYC in drinking water significantly decreased the frequency of radiation-induced (at doses of 1 Gy, 2 Gy and 4 Gy) MN and chromosomal aberrations in cultured human lymphocytes in vitro as compared to γ-irradiation alone (Srinivasan et al. 2009). Similarly, pre-treatment of human lymphocytes in vitro with various concentrations of LYC (10, 20, 40 µM/ml) directly and 1 h before irradiation with 1 Gy or 2 Gy decreased the DNA damage compared to the cells irradiated alone (Gajowik and Dobrzyńska 2017). LYC administration before diagnostic radiation exposure might be, therefore, useful to prevent damage to normal cells of cancer patients.

In the present study, only LYC at 0.15 mg/kg bw administered with a 24-h delay following radiation exposure mitigated the effect of irradiation at a dose of 1 Gy, both in peripheral blood and bone marrow RET. In contrast, a higher dose of 0.30 mg/kg bw LYC combined with irradiation induced a slightly increased level of MN in the bone marrow. In the case of 0.5 Gy radiation dose, the results of combined exposure were not significantly different compared to those of irradiation alone. No effects on MN frequency were observed in the case of LYC administration initiated on the eighth day following the first radiation exposure. These results imply that only the lower dose of LYC is protective against damage up to a certain level of fractionated radiation exposure, demonstrating a potential radiation threshold for protection. Generally, application of LYC before irradiation seems to be more beneficial, but not always is its effect on the radiation effect predictable. This study also analyzed the impact of LYC supplementation after the start of irradiation. Ability of LYC to decrease radiation-induced damage might be useful in the case of radiological accidents as well as possibly for radiological protection of non-treated tissues of cancer patients.

The LYC doses in the current paper are based on the daily intake that people may consume via dietary supplements (0.15 or 0.30 mg/kg bw, which corresponds to 10.5 or 20.1 mg/day for adults weighing 70 kg). The average daily intake of LYC in the United States ranges from 6.6 to 10.5 mg/day for men and from 5.7 to 10.4 mg/day for women, whereas in the United Kingdom it is 1.1 mg/day, in Spain 1.6 mg/day, in Australia 3.8 mg/day, in France 4.8 mg/day, and 4 in the Netherlands 9 mg/day (Porrini and Riso 2005). Based on the results of the present study, it may be concluded that the intake level of LYC may be sufficient to reduce radiation effects among US citizens, but too low for inhabitants of Europe and Australia.

It is well-established that the antioxidant properties of carotenoids are related to their radical scavenging properties as well as their exceptional singlet oxygen-quenching abilities (Conn et al. 1991; Hill et al. 1995; Everett et al. 1996; Mortensen and Skibsted 1996). As reported previously, in addition to antioxidant properties, carotenoids can also act as pro-oxidants, especially at higher concentrations (Miller et al. 1996; Martin et al. 1999; El-Agamey et al. 2004). The related antioxidant/prooxidant properties are likely to depend on factors like the rate of scavenging different types of radicals, the mode of reaction, and the properties of the resulting carotenoid radicals (Everett et al. 1996; Martin et al. 1999; Rice-Evans et al. 1997). The above finding may explain our results, where a dose of 0.15 mg/kg bw LYC applied with a 24-h delay mitigated the induction of MN by 1 Gy, whereas a higher dose, i.e., 0.30 mg/kg bw LYC, significantly enhanced the induction of MN. Young and Lowe (2001) proposed that the decreased antioxidant effect is due to carotenoid aggregation. Another explanation is a faster rate of carotenoid anti-oxidation (Palozza 1998). Forssberg et al. (1959) reported a moderate curative action (protection from lethal bacterial infection) with an increased survival rate with LYC administration both before and after X-ray irradiation in cancer-bearing mice in vivo. In an in vitro study, Cavusoglu and Yalcin (2009) observed a protective effect of LYC on radiation-induced chromosomal aberrations in lymphocytes when applied before irradiation. Moreover, DNA damage induced in human lymphocytes with H_2_O_2_ in vitro was significantly reduced after the subjects consumed a tomato-rich diet (Duthie et al. 1996; Viuda-Martos et al. 2014). Similarly, the frequency of MN induced in polychromatic erythrocytes of bone marrow of mice in vivo by benzo[*a*]pyrene or cyclophosphamide was significantly reduced by LYC (Rauscher et al. 1998).

As shown in previous in vivo studies, LYC may protect cells against radiation toxicity, predominantly through an antioxidant pathway (Srinivasan et al. 2007, 2009; Andic et al. 2009; Saada et al. 2010; Meydan et al. 2011). Srinivasan et al. (2009) showed that pre-treatment with LYC protects lymphocytes from γ-radiation-induced damage by inhibiting the peroxidation of free radicals and DNA strand breaks. Jomova et al. (2012) noted that LYC at a concentration of 30 µg/g (i.e., 30 mg/kg) acts as a prooxidant. LYC significantly decreased the level of thiobarbituric acid-reactive substances (TBARS) induced by ferric nitrilotriacetate (Fe/NTA), but enhanced the level of TBARS induced by a lipid-soluble radical generator (2,2-azobis[2,4-dimethylvaleronitrile]) (AMVN) (Yeh and Hu 2000).

In conclusion, our study showed that lycopene may act as a radiomitigator but only when applied at a low concentration 24 h, but not 8 days after irradiation. LYC may be used in clinical practice to protection of healthy tissues of radiology patients as well as in the case of radiological emergency. Contrary, combined exposure with high doses of irradiation and LYC may enhance the mutagenic effect of irradiation.
